# Changes in smoking prevalence among U.S. adults by state and region: Estimates from the Tobacco Use Supplement to the Current Population Survey, 1992-2007

**DOI:** 10.1186/1471-2458-11-512

**Published:** 2011-06-29

**Authors:** Ahmedin Jemal, Michael Thun, Xue Q Yu, Anne M Hartman, Vilma Cokkinides, Melissa M Center, Hana Ross, Elizabeth M Ward

**Affiliations:** 1 Intramural Research, American Cancer Society, 250 Williams Street NW, Atlanta, GA 30303, USA; 2 The Cancer Council New South Wales, 153 Dowling Street, Woolloomooloo, NSW 2011, PO Box 572, Kings Cross NSW 1340, Australia; 3 Cancer Control and Population Sciences, National Cancer Institute, 6130 Executive Boulevard, Rockville, MD, 20852, USA

## Abstract

**Background:**

Tobacco control policies at the state level have been a critical impetus for reduction in smoking prevalence. We examine the association between recent changes in smoking prevalence and state-specific tobacco control policies and activities in the entire U.S.

**Methods:**

We analyzed the 1992-93, 1998-99, and 2006-07 Tobacco Use Supplement to the Current Population Survey (TUS-CPS) by state and two indices of state tobacco control policies or activities [initial outcome index (IOI) and the strength of tobacco control (SOTC) index] measured in 1998-1999. The IOI reflects cigarette excise taxes and indoor air legislation, whereas the SOTC reflects tobacco control program resources and capacity. Pearson Correlation coefficient between the proportionate change in smoking prevalence from 1992-93 to 2006-07 and indices of tobacco control activities or programs was the main outcome measure.

**Results:**

Smoking prevalence decreased from 1992-93 to 2006-07 in both men and women in all states except Wyoming, where no reduction was observed among men, and only a 6.9% relative reduction among women. The percentage reductions in smoking in men and women respectively were the largest in the West (average decrease of 28.5% and 33.3%) and the smallest in the Midwest (18.6% and 20.3%), although there were notable exceptions to this pattern. The decline in smoking prevalence by state was correlated with the state's IOI in both women and men (r = -0.49, p < 0.001; r = -0.31, p = 0.03; respectively) and with state's SOTC index in women(r = -0.30, p = 0.03 0), but not men (r = -0.21, p = 0.14).

**Conclusion:**

State level policies on cigarette excise taxes and indoor air legislation correlate strongly with reductions in smoking prevalence since 1992. Strengthening and systematically implementing these policies could greatly accelerate further reductions in smoking.

## Background

Tobacco use remains the single largest preventable cause of disease and premature death in the United States. About 20% (46 million) of US adults are current smokers [1]. Tobacco use increases the risk of many diseases including heart disease, cancer, and respiratory diseases. Each year smoking results in an estimated 443,000 premature deaths, of which about 49,400 are in nonsmokers as a result of exposure to secondhand smoke [2]. In recent years, progress in the reduction of smoking and smoking related diseases varies by state.

Recent reports based on 1998 to 2007 Behavioral Risk Factor Surveillance System (BRFSS) surveys qualitatively showed that the prevalence of smoking generally is higher and the annual percentage decrease is lower among states in the South or Midwest, compared to those in the West or North East [3-5]. An evaluation of the American Stop Smoking Intervention Study (ASSIST) [6,7] observed larger reductions in per capita cigarette consumption and to a lesser extent smoking prevalence in states that implemented stronger policy and program interventions than in those that implemented weaker ones. The aim of ASSIST was to demonstrate that the application of statewide tobacco prevention and control programs and policies would reduce cigarette consumption and smoking prevalence. The ASSIST program which began in 1992-93 and concluded in 1998-1999 sought to change the social and environmental influences that affect individuals' use of tobacco, primarily through interventions in four policy areas: 1) smoke-free environments, 2) tobacco advertising and promotion, 3) youth access to tobacco, and 4) tobacco price [7]. The studies that evaluated ASSIST either did not systematically evaluate the changes in smoking prevalence in relation to state tobacco control policies and programs [3-5] or were limited to observations through 1998-99 [6,7]. Several other analyses [8-15] have observed accelerated reductions in per capita consumption and/or smoking prevalence in states or cities that have implemented major increases in cigarette excise taxes and other elements of comprehensive tobacco control programs.

This paper examines changes in state- and regional adult smoking prevalence from 1992-2007 in relation to two indices of state tobacco control policies measured in 1998-99, with longer follow-up than previous analyses (ASSIST) allowing greater time for policy interventions and programs to have an effect. The two indices of tobacco control policies measured in 1998-99 were the Initial Outcome Index (IOI) which measures states' tobacco control policies and activities (such as indoor air legislation and cigarette prices) and the strength of tobacco control (SOTC) index which measures tobacco control resources, capacity, and program efforts [16,17]. In addition to examining the extent to which the indices of state tobacco control policies explain the trends, we also identify notable exceptions (outliers) that deserve further scrutiny.

## Methods

This study uses current smoking prevalence data from three waves of Tobacco Use Supplement to the Current Population Survey (TUS-CPS), 1992-93, 1998-99 and 2006-07, each with a three month sample. The CPS is a monthly survey of over 50,000 households conducted by the Bureau of the Census for the Bureau of Labor Statistics [18]. It is a probability sample based on a stratified sampling scheme of clusters designed to provide representative estimates for the whole nation, regions, and individual states. The complete CPS methodology has been published elsewhere [19]. The Tobacco Use Supplement (TUS) has been administered periodically as part of the CPS since the 1992-93 CPS [20] to measure current smoking and other measures of tobacco use nationally and by region and state. Current smokers were defined as those who smoked every day or some days and had smoked at least 100 cigarettes in their life time.

We estimated weighted current smoking prevalence for ages 18 and older by sex and area of residence (state, division and region) for 1992-93, 1998-99, and 2006-07, separately, by use of TUS-CPS survey weights (which account for selection probabilities and survey non-response). Then we computed the relative percentage change in smoking prevalence in 2006-07 compared to 1992-93, i.e., the absolute difference in smoking prevalence between 2006-07 and 1992-93 expressed as a percentage of the 1992-93 prevalence. The statistical significance of this relative change was assessed by examining whether the ninety-five percent confidence intervals (95% CIs) of the two smoking proportions overlapped or not. Standard errors for the weighted prevalence were computed in SAS-callable SUDAAN (V.9.0.1) [21], using PROC CROSSTAB, with replicate weights obtained from the U.S. National Cancer Institute (NCI), with Fay's balanced repeated replication [22].

We determined whether the relative changes in smoking prevalence were associated with summary measures of state tobacco control policies or activities in 1998-1999, specifically the initial outcome index (IOI) and the strength of tobacco control (SOTC) index, both of which were developed to assess the effectiveness of the ASSIST program. The IOI was designed to serve as a near-term measure for the effectiveness of ASSIST interventions in order to capture societal changes that are fostered by early intervention strategies that will ultimately, over time, result in the final desired outcomes such as lower smoking prevalence and per capita cigarette consumption. The IOI was formed from three initial outcomes, each of which was significantly correlated with reduced prevalence and consumption levels at baseline in 1993: the percentage of smokers reporting working in a 100% smoke-free work site, price of cigarettes, and legislative rating for clean-indoor air which is a score that reflects both the strictness and the coverage of clean air ordinances within each state. An example of a clean air ordinance is the California state law enacted in 1995 that prohibited smoking in nearly all indoor workplaces, which was extended in 1998 to include bars and gaming rooms. The data for these outcomes were obtained from national datasets such as the TUS-CPS and the State Cancer Legislative Database (SCLD) and from local data from the American Nonsmokers' Rights Foundation (ANRF). The IOI index value was formed by creating z scores (standardized values) by state for each of the three tobacco control measures, which were then summed to form an overall index for tobacco control efforts in each state [7]. The SOTC index was created to measure the program effects of ASSIST and to serve as an overall measure of tobacco control intensity at the state level. The measure comprises three main constructs: 1) tobacco control resources which were defined as the amount of money allocated for a state's tobacco control program and the number of full-time equivalent staff assigned to tobacco control in a state, 2) capacity which was defined as state leadership support for tobacco control, the character of relationships between state tobacco control agencies, the independence and power of the health department tobacco control program director, the composition and character of the state-level tobacco control coalition(s), and the experience level of state tobacco control professionals, and 3) program efforts focused on policy and environmental changes defined by the tobacco control activities that the state tobacco control program engaged in such as media advocacy efforts to gain anti-tobacco coverage and education and cessation programs [7]. The constructs were measured through a survey instrument with respondents from a variety of sources including state health departments, statewide tobacco control coalitions, and state level voluntary health organizations. The SOTC index values were created through the use of z scores sums and a hierarchical principal components analysis [6,7,16,17].

The relationship between these two indices and changes in smoking prevalence were evaluated using Pearson partial correlation coefficients after accounting for state differences in percent poverty, or percent black or Hispanic according to the 2000 U.S. Census. The IOI has been measured during in 1992-93 and 1998-99 and in each year in between; we chose to use the IOI measured in 1998-99 because it reflects the overall strength of tobacco control policies and efforts at the midpoint of the period in which trends in smoking prevalence were examined. The SOTC index was measured for the first time in 1998-1999 [17].

Because many states have increased the price of cigarettes after 1998-99 and/or implemented new or strengthened existing tobacco control programs following the Master Settlement Agreement in 1998-1999, we also calculated state-specific changes in smoking prevalence between 1998-99 and 2006-07 and their associations with state-specific changes in inflation adjusted price of cigarettes (1998-99-2005) or in percent of indoor workers with smoke-free work place during the corresponding time interval (1998-2007).

We mapped the smoking prevalence in 2006-07 and the relative percent change in the prevalence between 1992-93 and 2006-07 by state to illustrate the top 10 and bottom 10 states and regional patterns for each of these two variables. We restricted our main analyses to these two time periods because we were interested in the total percent change between the two time intervals.

## Results and Discussion

**Figure 1:**
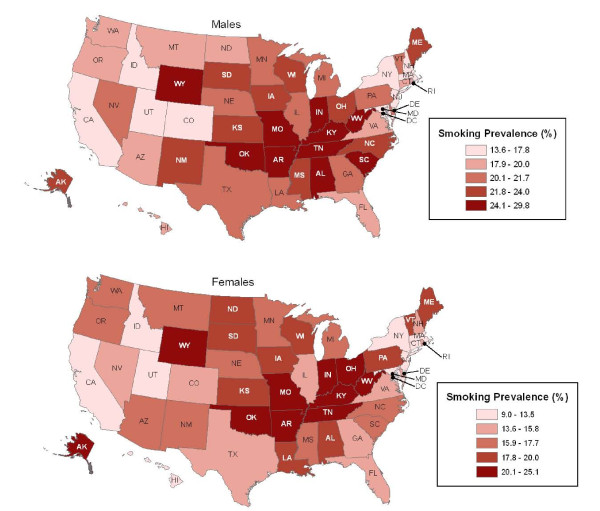
**Smoking prevalence by sex and state, 2006-2007**. Source: Current Population Survey, 2006/07

Table 1 shows current smoking prevalence among adults aged 18 years and older by state for men and women for the 1992-93, 1998-99, and 2006-07 surveys. During the 2006-07 survey, the highest smoking prevalence was recorded in Kentucky for men and in Kentucky and West Virginia for women. Seven of the top ten states for smoking prevalence in men and five of the top ten states in women are located in the Southern region (Figure 1). In contrast, Utah showed the lowest smoking prevalence for men and Utah and California for women during the corresponding time interval.

**Table 1:** Current smoking prevalence among adults (aged 18 years or older) by sex and state, according to data from Current Population Survey, 1992-1993, 1998-1999, and 2006-2007

	Males							Females								
			
State	1992-93		1998-99		2006-07		Relative Change (%) 1992-93 vs. 2006-07*	1992-93		1998-99		2006-07		Relative Change (%) 1992-93 vs. 2006-07*	IOI	SOTC
		
	Prop (%)	SE	Prop (%)	SE	Prop (%)	SE		Prop (%)	SE	Prop (%)	SE	Prop (%)	SE			
Alabama	29.7	0.83	24.6	1.39	24.1	1.38	-19.0†	22.2	0.88	20.2	1.19	18.6	1.39	-15.9	0.89	-0.18
Alaska	29.0	1.84	27.0	2.64	24.1	1.08	-17.1	26.3	1.35	26.4	1.39	21.3	1.04	-18.9†	10.55	0.30
Arizona	24.7	1.17	22.3	1.08	19.2	0.99	-22.3†	20.1	1.16	17.6	0.88	16.1	1.00	-20.1	5.25	4.03
Arkansas	32.6	1.55	28.1	1.55	26.7	1.70	-18.2	25.3	1.16	24.1	1.40	21.4	1.43	-15.4	1.99	0.08
California	22.4	0.46	19.7	0.60	15.2	0.45	-32.2†	15.6	0.43	13.6	0.45	9.0	0.33	-42.2†	6.74	3.73
Colorado	25.3	1.07	21.1	0.95	17.8	1.01	-29.8†	23.1	1.12	19.3	0.89	14.7	0.82	-36.2†	2.75	-0.40
Connecticut	24.2	1.41	22.5	2.03	17.9	0.97	-26.1†	20.6	1.02	18.7	1.42	13.1	0.68	-36.2†	4.22	0.37
Delaware	24.3	1.45	23.8	1.38	20.1	1.23	-17.4	22.3	1.77	22.9	1.43	15.7	0.98	-29.5†	2.07	-1.07
District of Columbia	26.1	1.25	26.6	1.75	17.0	1.22	-34.8†	20.9	1.18	21.0	1.32	13.5	0.99	-35.3†	6.85	-0.87
Florida	26.7	0.71	23.8	0.74	18.0	0.73	-32.3†	21.4	0.60	17.9	0.53	13.7	0.59	-36.1†	3.53	1.70
Georgia	28.3	1.70	23.9	1.30	21.0	1.21	-26.0†	20.8	1.34	16.4	0.85	15.1	0.87	-27.5†	1.73	0.39
Hawaii	25.9	1.43	22.0	1.34	18.6	1.14	-28.1†	18.8	1.25	15.1	1.17	12.3	0.87	-34.3†	9.04	0.96
Idaho	26.2	1.29	24.7	1.70	16.9	1.18	-35.7†	21.1	1.42	18.8	1.57	13.4	1.16	-36.2†	3.78	0.13
Illinois	27.1	0.75	25.1	0.80	20.9	0.86	-23.0†	22.1	0.61	20.9	0.54	15.8	0.79	-28.5†	4.61	-0.71
Indiana	31.6	2.06	30.3	1.52	26.8	1.33	-15.3	24.6	1.06	24.1	1.22	22.1	1.11	-10.1	1.42	-1.08
Iowa	26.5	0.71	25.1	1.29	23.1	1.11	-12.9	20.6	1.38	19.7	1.12	19.1	1.03	-7.0	2.17	0.41
Kansas	25.0	1.40	24.4	1.45	22.9	1.31	-8.3	23.5	1.58	20.7	1.26	17.9	0.90	-23.8†	4.89	0.47
Kentucky	35.8	1.19	32.4	1.42	29.8	1.36	-16.7†	28.5	1.26	27.5	1.34	25.1	1.10	-11.8	-1.09	-0.19
Louisiana	27.2	1.81	27.3	1.42	21.5	2.09	-21.0	23.8	1.36	18.8	0.97	18.4	1.36	-22.5	2.64	-2.30
Maine	30.5	1.47	25.6	1.67	23.5	1.09	-22.8†	26.6	1.19	21.6	1.32	19.1	0.97	-28.2†	6.96	-1.24
Maryland	24.1	1.03	22.1	1.34	17.8	0.89	-26.3†	23.1	1.50	17.1	1.08	13.5	0.90	-41.7†	8.24	0.97
Massachusetts	22.1	0.74	22.2	1.11	16.6	1.12	-24.9†	20.8	0.71	16.7	0.81	12.9	0.98	-38.1†	8.63	0.46
Michigan	29.8	0.75	25.1	0.79	21.4	0.95	-28.2†	24.7	0.65	21.7	0.85	17.5	0.84	-29.4†	6.64	0.90
Minnesota	26.3	1.47	22.7	1.08	20.9	1.08	-20.3†	24.1	1.54	19.8	1.19	16.0	0.73	-33.5†	5.96	1.74
Mississippi	30.3	1.41	26.2	1.42	23.9	1.50	-21.3†	21.0	1.12	17.6	1.23	16.7	1.33	-20.5	0.76	1.28
Missouri	28.3	1.57	26.1	1.43	26.7	1.41	-5.6	24.4	1.76	21.1	1.28	20.9	1.04	-14.5	3.38	-0.79
Montana	23.0	1.36	23.4	1.29	18.8	1.30	-18.0	24.1	1.15	23.2	1.20	16.9	1.33	-29.8†	2.88	-1.60
Nebraska	24.1	1.02	21.8	1.29	20.9	0.93	-13.4	20.0	1.19	20.6	0.81	17.2	0.99	-14.0	3.61	-0.31
Nevada	29.7	1.12	25.6	1.38	20.8	1.22	-29.8†	27.1	1.07	22.5	1.23	15.3	1.16	-43.4†	1.27	-1.42
New Hampshire	24.9	1.30	23.5	1.50	17.4	0.86	-30.4†	24.5	1.47	20.7	1.22	15.8	0.87	-35.5†	5.42	-0.45
New Jersey	21.1	0.66	21.3	0.85	15.4	0.87	-26.8†	19.7	0.58	18.6	0.85	11.1	0.76	-43.9†	7.93	1.12
New Mexico	27.5	1.34	24.1	1.16	22.8	1.42	16.9	20.7	1.09	18.0	1.29	15.9	1.58	-23.1	2.70	-0.53
New York	23.5	0.51	23.0	0.63	17.3	0.62	-26.3†	19.8	0.57	18.4	0.59	13.0	0.52	-34.2†	8.03	0.69
North Carolina	31.7	0.93	25.9	0.94	23.3	1.04	-26.5†	22.6	0.64	20.3	0.73	16.7	0.77	-26.2†	0.41	-0.14
North Dakota	23.0	1.48	21.7	1.99	19.8	1.25	-13.9	21.4	1.96	19.3	1.15	18.3	1.13	-14.6	5.04	-0.93
Ohio	28.1	0.53	24.9	0.94	23.3	0.88	-17.0†	24.1	0.65	23.2	0.80	21.7	1.01	-9.8	2.40	-1.05
Oklahoma	29.1	1.45	30.3	0.95	25.5	1.46	-12.5	24.7	1.42	25.1	0.90	21.8	1.48	-11.8	1.46	0.84
Oregon	26.4	1.13	23.3	1.30	20.0	1.21	-24.4†	19.5	0.95	19.1	1.03	15.9	0.86	-18.6†	4.96	0.90
Pennsylvania	25.4	0.71	25.0	0.67	20.4	0.71	-19.7†	21.5	0.61	21.0	0.68	17.9	0.75	-16.7†	1.79	-0.68
Rhode Island	22.0	1.00	21.5	1.40	18.9	0.97	-14.3	24.2	1.01	18.4	1.14	15.3	0.77	-37.0†	6.88	1.09
South Carolina	29.6	1.19	26.1	1.39	24.6	1.23	-17.0†	22.3	1.09	19.9	1.36	17.6	1.10	-21.0†	0.47	-0.48
South Dakota	28.5	1.39	25.5	1.77	22.4	1.14	-21.5†	22.8	1.07	22.7	1.36	18.1	1.25	-20.5	0.75	-1.20
Tennessee	32.3	1.61	29.8	1.56	26.1	1.60	-19.2	25.4	1.05	22.5	1.09	21.2	1.18	-16.5	0.58	-1.28
Texas	27.1	0.82	24.3	0.69	20.1	0.69	-26.1†	20.0	0.58	17.7	0.57	13.9	0.43	-30.4†	3.75	-0.61
Utah	20.2	1.12	16.4	1.31	13.6	1.13	-32.7†	13.8	0.95	11.4	1.11	10.6	1.16	-23.4	7.77	-0.29
Vermont	29.2	1.44	23.9	1.38	20.8	1.20	-28.6†	23.5	1.73	20.9	1.07	19.1	1.17	-19.1	6.37	-1.15
Virginia	29.5	1.47	24.1	0.93	18.1	1.01	-38.6†	21.8	1.26	17.7	0.86	14.8	0.88	-32.1†	1.44	0.07
Washington	25.9	1.22	21.8	1.13	18.9	0.98	-26.9†	22.5	1.16	18.6	0.99	16.6	1.04	-26.5†	8.45	0.23
West Virginia	32.6	1.50	28.9	1.46	25.1	1.45	-23.1†	28.1	1.50	24.2	1.59	25.1	1.07	-10.7	1.77	-0.53
Wisconsin	27.0	1.26	28.8	1.43	21.8	1.03	-19.2†	25.3	1.62	20.3	1.34	20.0	0.84	-21.1†	5.74	-0.04
Wyoming	24.9	1.32	23.9	1.35	24.9	1.06	0.0	24.8	1.10	23.5	1.24	23.0	1.12	-6.9	0.33	-0.92
Total United States	26.5	0.17	24.0	0.15	20.1	0.16	-24.2†	21.5	0.16	19.0	0.13	15.5	0.12	-27.6†		

*The absolute difference is smoking prevalence between 2006/7 and 1992/3 as expressed as a percentage of the 1992-93 and 1998-99 prevalence, respectively.

† The 95% confidence intervals for 1992-1993 and 2006-2007 prevalence estimates do not overlap.

IOI = Initial Outcome Index measures the percentage of smokers covered by 100% smoke-free work sites, price of cigarette, and legislative rating for clean-indoor air. The IOI was measured in 1998-99 in order to assess the effectiveness of the ASSIST program.

SOTC = Strength of Tobacco Control index measures tobacco control resources, capacity and program efforts focused on policy and environmental changes. The SOTC was measured in 1998-99 in order to assess the effectiveness of the ASSIST program.

**Figure 2:**
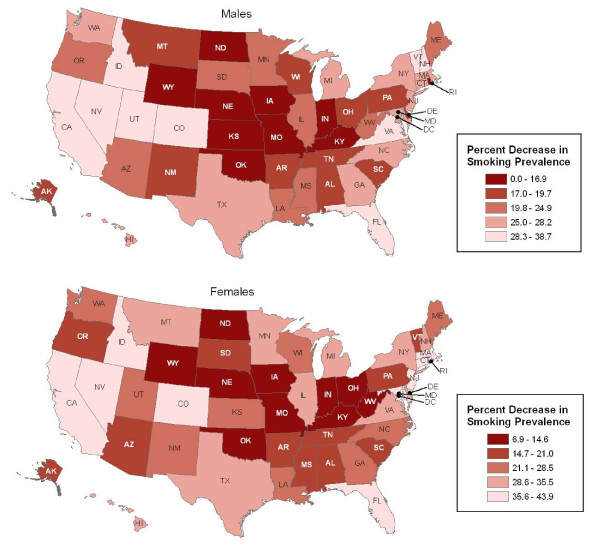
**Relative change in smoking prevalence between 1992-1993 and 2006-2007 by state**. Source: Current Population Survey, 1992/93 and 2006-07

Adult smoking prevalence decreased from 1992-93 to 2006-07 in both men and women in all states but Wyoming (Table 1). States with the largest percentage reduction among men were Virginia (38.6%), Idaho (35.7%), Washington DC (34.8%), Utah (32.7%), and Florida/California (both 32.3%) (Table 1, Figure 2). Those with the largest percentage reduction among women were New Jersey (43.9%), Nevada (43.4%), California (42.2%), Maryland (41.7%) and Massachusetts (38.1%). States with the smallest percentage reduction among men were Wyoming (0%), Missouri (5.6%), Kansas (8.3%), Oklahoma (12.5%) and Iowa (12.9%), and among women they were Wyoming (6.9%), Iowa (7.0%), Ohio (9.8%), Indiana (10.1%) and West Virginia (10.7%).

Regionally, the percentage reductions in smoking from 1992-93 to 2006-07 among men and women respectively were the largest in the West (average decrease of 28.5% and 33.3%) and the smallest in the Midwest (18.6% and 20.3%) (Additional File 1, Table S1). Within region, the decreases by state were not statistically significant in 8 of 12 mid-western states and 9 of 17 southern states for at least one sex. Seven mid-western states (North Dakota, Nebraska, Iowa, Kansas, Missouri, Indiana, and Ohio) were in the bottom 10 states for reductions in male and/or female smoking (Figure 2).

Considerable heterogeneity in the trends was observed in certain regions, especially in the Mountain States and Midwest (Additional File 1, Table S1 and Figure 2). Whereas Wyoming had the smallest percentage decrease in both sexes, the bordering state of Idaho had the second largest percentage decrease in men (35.7%) and was in the top quintile for women (36.2%). South Dakota had greater reductions in male and female smoking (by 21.5%, 20.5%) respectively than the neighboring states of either North Dakota (13.9%, 14.6%) or Nebraska (13.4%, 14.0%). Similarly, the reductions in male and/or female smoking, respectively, were much smaller in Missouri (5.6%, 14.5%), Iowa (12.9%, 7.0%), and Kansas (8.3%, 23.8%) than in Michigan (28.2%, 29.4%), and Minnesota (20.3%, 33.5%).

The decrease in smoking prevalence by state was not strongly correlated with the 1992-93 baseline smoking prevalence in either women (r = 0.29, p = 0.04) or men (r = 0.10, p = 0.47). For example, the relative percentage reduction in women's smoking prevalence was as large in California (42.2%) as in Nevada (43.4%), even though the initial smoking prevalence was nearly half as high among women in California. Further, the relative percentage decrease in smoking prevalence was generally greater in women (median = 23.4%) than in men (median = 21.5%), while the relative prevalence of smoking was higher in men than women in all states except Rhode Island (22.0%, 24.2%) and Montana (23.0%, 24.1%) at the start of the study.

**Figure 3:**
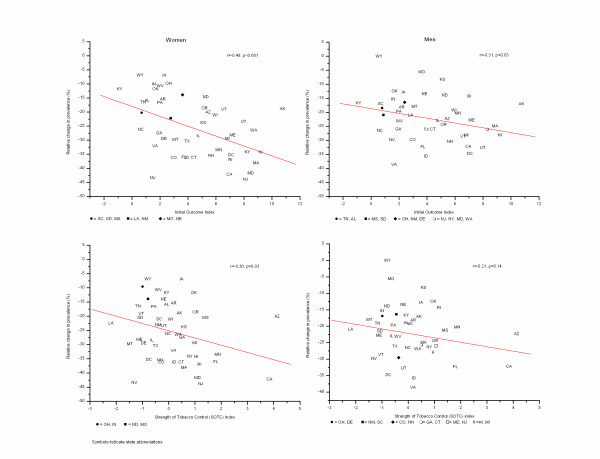
**Relationship between changes in smoking prevalence and indices of state tobacco control policies and activities (IOI) and tobacco control resources and capacity (SOTC)**.

The relative change in smoking prevalence by state was significantly correlated with the state's IOI score in both women (r = -0.49, p < 0.001) and men (r = -0.31, p = 0.03) and with state's SOTC index in women (r = -0.30, p = 0.03), but not in men (r = -0.21, P = 0.14) (Figure 3). The correlation coefficients remained unchanged when we adjusted for percent federal poverty level and/or percent black in the population. They were not also affected by exclusion of states with extreme values or outliers, including Arizona and California for the highest IOI, Alaska for the highest SOTC, and Wyoming and Nevada for the lowest and highest relative changes, respectively. However, the associations became weaker or non-existent when we restricted the analysis to non-Hispanic whites (r = -0.2 to r = 0.01, p > 0.1), except for IOI among women (r = -0.54, p < 0.0001).

In contrast to the 1992-93 to 2006-07 period where smoking prevalence statistically decreased in 35 states in men and 31 states in women, smoking prevalence from 1998-99 to 2006-07 significantly decreased in 14 states in men and 19 states in women, with 5 of these states in men and 4 in women located in the North East (Additional File 2, Table S2). Overall the decreases in the smoking prevalence from 1998-99-2006-07 by state were not statistically significantly correlated with changes in the price of cigarettes (men, r = -0.1, p = 0.5; women, r = -0.2, p = 0.13), nor with changes in percent of indoor workers with smoke-free work place (men, r = -0.2, p = 0.18; women, r = 0.0, p = 0.8) over the corresponding time interval.

## Conclusions

Tobacco control policies at the state level have been a major impetus for reductions in smoking prevalence in the United States. We observed that states with higher indices of tobacco control, and specifically measured by cigarette excise taxes, smoke-free work places and legislation (IOI), had larger reductions in smoking prevalence over the 15 years of observation than states with weaker policies. These results suggest that cigarette taxes and smoke free laws may have a more direct effect on tobacco control than does SOTC, which reflects programmatic resources and capacity. It could be that there needs to be a threshold of SOTC that must be maintained to give rise to the policies that directly impact smoking prevalence [23]. Increased excise taxes on cigarettes and restrictions on smoking in work places have been shown to reduce smoking prevalence and consumption [24-26].

Our results extend the findings of Stillman et al. [6] by providing an additional eight years of observation since the tobacco control measures were implemented. Our results, like those of Stillman et al. [6], found a greater reduction in smoking prevalence associated with the IOI in women than in men. Whether this reflects higher price sensitivity and/or greater responses to other factors that track with policy changes, such as media campaigns on the health hazard of smoking in women, is not known. Similar to our findings, Stillman et al. did not find an association between SOTC and reduction in smoking prevalence [6].

We observed prominent regional variation in smoking prevalence and in the reductions in prevalence as reported previously by others [3,4,27,28]. The Midwest experienced the smallest reduction in smoking prevalence in both men and women (18.6% and 20.3% respectively); the West, influenced strongly by California, had the largest reduction for both men (28.5%) and women (33.3%). Intermediate patterns were seen in the South (reductions of 25.5% in men and 26.9% in women) and North East (24.4% and 31.7%). It is interesting that the observed relative percentage reduction in smoking prevalence was more closely correlated with tobacco control measures, particularly the IOI, than with the initial smoking prevalence in the state. This suggests that strengthening state tobacco control programs should further reduce smoking prevalence in all states, even in those with already low smoking prevalence such as California and Utah.

There is considerable heterogeneity within region in reduction of smoking prevalence. For example, whereas Wyoming had the smallest relative percentage decrease in both sexes, the bordering state of Idaho had the second largest relative percentage decrease in men and was in the top quintile for women. Some of this heterogeneity is related to variations in tobacco control policies [5,29,30], but other factors are clearly influential in certain states. The large reduction in Nevada, particularly among women, may in part reflect recent influx of Hispanic immigrants into this state [31]. In general, smoking prevalence is lower in foreign born Hispanics than US born Hispanics [32,33]. The large decrease in North Carolina, Virginia, and New Jersey may reflect changes in the economic base or proximity to major metropolitan areas that likely influence the trends. However, it is not clear why smoking prevalence in Arkansas has decreased more slowly than would be predicted from the two indices of tobacco control.

The TUS-CPS survey uses consistent study design and interview methodology across surveys and states/regions for comparisons of prevalence data over time and across regions. In addition, the survey has a high response rate, about 88% for the 1992-93 survey and 83% for the 2006-07 survey. This contrasts with about a 50% or lower response rate for the BRFSS [27,34]. Our main findings were not affected by the choice of correlation method (Pearson vs. Spearman) or by the exclusions of outliers.

There are a number of limitations in the data and analysis that may affect the interpretations of our findings. About 75% of interviews in the 1992-93 TUS-CPS and 64% of interviews in the 2006-07 survey were conducted by telephone rather than in person. However, this percentage varied very little across states for both the 1992-93 and the 2006-07 surveys. For the 2006-07 survey, the percentage of telephone interviews ranged from 50% in Florida to about 70% in Vermont. A second limitation of TUS-CPS is that about 18% of responses in the 1992-93 survey and 24% of responses in the 2006-07 survey were proxy rather than self-reported. However, self-responses and proxy responses yield comparable estimates for adult smoking prevalence [28] except perhaps for young adults [35].

Third, in our correlation analysis we did not account for tobacco industry activities that primarily target states with strong tobacco control programs because these data are not readily available [36,37]. This may have attenuated our findings. Changes over time by geographic area can be affected by mobility. However, the effect of migration is considered to be minimal when the unit of analysis is a large geographic area such as state as opposed to a small geographic area such as a county or a census tract [38].

Fourth, while the most important confounders (inflation-adjusted cigarette prices and percent of indoor workers covered by smoke-free workplaces) were controlled for in our analyses it is possible that residual confounding from tobacco control policies implemented after 1998-99 that generally have immediate effects on behavior, such as public smoking bans, may have contributed to the lack of association between 1998-99 the IOI and SOTC and 2006-07 smoking prevalence.

A recent analysis of smoking data from the National Health Interview Survey showed that the decrease in smoking prevalence at the national level has stalled from 2007 to 2008 [1]. Future studies should examine the extent of state variations in this more recent pattern when data become available.

In conclusion, state level policies on cigarette excise taxes and indoor air legislation correlate strongly with reductions in smoking prevalence since 1992. The wide variations in progress, even among neighboring states, suggest that strengthening and systematically implementing these policies could greatly accelerate further reductions in smoking.

## Competing interests

The authors declare that they have no competing interests.

## Authors' contributions

AJ conceived of the study, participated in its design and coordination and helped draft the manuscript. MT, AMH, VC, HR, and EMW participated in study design and helped draft the manuscript. XQY and MMC participated in the design of the study and performed the statistical analysis. All authors read and approved the final manuscript.

## Pre-publication history

The pre-publication history for this paper can be accessed here:

http://www.biomedcentral.com/1471-2458/11/512/prepub

## Supplementary Material

Additional File 1 **Table S1**: Current smoking prevalence among adults (aged 18 years or older) by sex, region, census division and state, according to data from Current Population Survey, 1992-1993 and 2006-2007 Click here for file

Additional File 2 **Table S2**: Current smoking prevalence among adults (aged 18 years or older), by sex, state, division and region Current population surveys 1998-99 and 2006-07 Click here for file
